# Sustaining Hope Within Entangled Accompaniments: Toward an Otherwise Clinical Ethnography and Critical Social Medicine

**DOI:** 10.1007/s11013-025-09897-5

**Published:** 2025-02-09

**Authors:** Matthew Hing, Salmaan Keshavjee

**Affiliations:** 1 Department of Anthropology, University of California, Los Angeles, CA 90095 USA; 2 Department of Global Health and Social Medicine, Harvard Medical School, Cambridge, MA 02138 USA; 3 Division of Global Health Equity, Brigham and Women’s Hospital, Boston, MA 02130 USA

**Keywords:** Clinical ethnography, Social medicine, Accompaniment, Liberatory solidarity

## Abstract

The series of papers in this special issue, “Ethnography of and in Clinical Formation: Poetics and Politics of Dual Subjectivity,” touch on several themes that are at the core of social medicine: the web of social structures and power relations that organize the risk and prematurity of disease and death, who gets care when and where, and what that care looks like and does within situated social worlds. As Levenson and Samra (this issue) describe in their contribution, social medicine turns on extending the field of medical action “beyond the clinical encounter” in order to visibilize how such encounters are “organized by wider regimes of governance and expertise, and broader geographies of care, abandonment and violence.” Writing from the “fractured habitus” as reported by Schlesinger (Doing and seeing: Cultivating a “fractured habitus” through reflexive clinician ethnography, Somatosphere, 2021) of clinician-ethnographers, the authors here witness and interrogate the nascent possibilities for more liberatory and autonomous forms of care within these otherwise determining regimes. They also expose the limits of traditional clinical ethnographic positioning through authors’ diverse participations within spaces of organized violence – indicating the need for a “new conceit” (Aboiil, this issue) of the clinical ethnographer/social medicine practitioner who is open to sitting in the trouble of a “complicity consciousness” (Sufrin, this issue) and the expanded fields of theorizing, action, and accompaniment that it makes possible.

## Introduction

The series of papers in this special issue, “Ethnography of and in Clinical Formation: Poetics and Politics of Dual Subjectivity,” touch on several themes that are at the core of social medicine: the web of social structures and power relations that organize the risk and prematurity of disease and death, who gets care when and where, and what that care looks like and does within situated social worlds. As Levenson and Samra (this issue) describe in their contribution, social medicine turns on extending the field of medical action “beyond the clinical encounter” in order to visibilize how such encounters are “organized by wider regimes of governance and expertise, and broader geographies of care, abandonment and violence.” Writing from the “fractured habitus” as reported by Schlesinger (Doing and seeing: Cultivating a “fractured habitus” through reflexive clinician ethnography, Somatosphere, 2021) of clinician-ethnographers, the authors here witness and interrogate the nascent possibilities for more liberatory and autonomous forms of care within these otherwise determining regimes. They also expose the limits of traditional clinical ethnographic positioning through authors’ diverse participations within spaces of organized violence – indicating the need for a “new conceit” (Aboiil, this issue) of the clinical ethnographer/social medicine practitioner who is open to sitting in the trouble of a “complicity consciousness” (Sufrin, this issue) and the expanded fields of theorizing, action, and accompaniment that it makes possible.

The roots of social medicine are multiple and diverse (Allende, 1939; Du Bois, 1899; Engels, 1845; Fanon, 1959; Tristan, 1843; Virchow, 1848), but these branches converge around a shared sociogenic approach to illness (Kalofonos, this issue): recognition of how social forces manifest in power gradients, political-economic structures, and practices of (un)freedom that produce or limit health and shape clinical practice. Similarly, all propose various solutions aimed at restructuring power relations – reframing social relations otherwise – as a means of improving collective health. Clinical ethnography, as a key methodology of social medicine, remains an essential instrument for charting and critiquing the social dynamics and power relations that affect health. The manuscripts in this series enrich this genealogy of engaged clinical ethnography through continued problematization of these same gradients and the structural milieus within which they function. Social medicine is also about critical recognition of where would-be health professionals themselves are located within these fields and relations of power; as the articles here demonstrate, the “insider-outsider status” (Karlin & Hodge, this issue) of clinician-ethnographers provides a generative position for situating oneself to feel the weight of modern medicine’s neoliberal and carceral entanglements as well as to glimpse emergent and unknown “otherwise” possibilities for care and praxis.

These manuscripts revolve around several important themes. The first is careful “observant-participation” (Sufrin, 2015) of the ways that *healthcare and clinicians have increasingly become appendages of carcerality and capitalism*. The coterminous nature of the medical industrial complex and prison industrial complex has long been interrogated by social scientists, clinicians, and community activists (Ben-Moshe, 2020; Clayton-Johnson, et al., 2021; Roberts, 2022), and the pieces here contribute to this urgent scholarship by describing viscerally and painfully how multiple spheres of medicine – psychiatry, addiction care, reproductive care – are deeply imbricated with neoliberal thinking and carceral logics. These relationships also shape “the material circumstances of [medical] training” described in Holmes’s piece and their implications for trainee’s truncated capacity for empathy and solidarity, socializing clinicians early on into subjectivities productive for the “carceral therapeutic state” (Sue, 2019). Medicine becomes part of a transplanting mechanism for changing social relations and creating a specific type of social order that is linked to punishment, liability, and different forms of state and social surveillance. Direct clinical practice in penal institutions (so-called “correctional health”) is the starkest example of this process, where clinicians cannot cure or stop the state-sanctioned violence of incarceration, and even the intimately documented micro-subversions and harm reduction efforts by structurally attuned jail clinicians like Sufrin, Buchbinder, and Sue come to feel like stabilizing efforts. It is in understanding these local moral worlds (Kleinman, 1992) that the structural forces/violence that defines who lives and who dies, come into stark relief.

What many of the manuscripts in this series share is a desire for an “abolitionist future” where carceral and/or neoliberal logics are “not quotidian and acceptable” (Sue, this issue). Through their *attentive descriptions of* (so-called) *states of exception* they hypervisibilize the porous boundaries and precarious construction of these carceral and/or neoliberal formations as well as the “latent possibilities and potentialities held within [them]”(Meek & Morales, 2022) for liberation. Karlin and Hodge, Stonington, et al., Aboii, and Knight all narrate in different ways the problematic making of “crises” (COVID-19, abortion restrictions, corpses) as well what theses crises themselves can allow: reconfigurations of care, expertise, empathy, risk, and solidarity. They lead us to ask: do states of exception harden the neoliberal and carceral orders or provide space for more liberatory, autonomous practices? The former is a frequent outcome, but the authors here linger their gaze on the latter; the perceived exceptionality of these moments can destabilize traditional epistemes and narratives, calling into frame new “onto-interpretative [matrices]” (Zigon, 2018) that (un)know and (un)do differently.

Crisis-induced “hallucination” (Stonington, et al., this issue) becomes a form of radical –albeit highly unstable— reimagination that temporarily unsettles expertise and protocolized ways of knowing/doing. It operates like an x-ray to expose the lesions in care delivery systems built on the perpetuation of power gradients and social control. For example, spatial and social control of highly surveilled medications like methadone is partially called into question in ways that expand patients’ and providers’ senses of what is safe and possible (Knight, this issue). The exigencies of heightened reproductive surveillance and criminalization generate hotlines for women’s health counseling that provide rare opportunities for clinicians to partake in a more liberating clinical praxis aligned with their political and professional values. Authors’ interrogation of these moments of so-called “exception” makes more visible “otherwises” (Meek & Morales, 2022) that are ever present and available, and clarifies the need for a social medicine that is always attuned to these possibilities regardless of the false temporal bounding of crisis (Aboii, this issue).

While it is tempting to linger in the immanent possibilities of these hopeful states of exception to neoliberal carceral medicine described in many of this issue’s pieces, doing so both overwrites and fails to reckon adequately with *clinician-ethnographers’ complicity vis-à-vis institutions that perpetuate forms of structural violence* inside and outside moments of crisis – the last theme we want to expand upon. Multiple authors in this issue meditate on whether and to what degree their sustained presence and participation in sites and relations of structural violence makes them complicit in the perpetuation of systems that harm their interlocutors. They describe formal and informal strategies for both sidestepping and confronting these entanglements during their work, practicing refusal and reimagination when possible but maintaining their gaze on the undeniable embroilment of their position. “Complicity consciousness,” as Sufrin suggests, takes as a starting point the fraught situatedness of clinicians and ethnographers within the systems they critique, and allows them to understand potential acts of (ac)complicity that in fact can *only* be taken from that critical positioning.

Recognizing the moral injury that results from complicity within systems of violence opens the door to different forms of accompaniment and makes different fields of action possible. These articles certainly demonstrate the fraught but fertile positioning of being “there,” how the insiderness of providing direct care and participating non-innocently alongside interlocutors can be a productive strategy to “uncloud” (Farmer, in Powell, 2018) structural vision and resocialize illness and care when a primary problem among clinicians is often “an inability to see what matters” (Kalofonos, this issue). However, clinician-ethnographers’ frequent roles as “witnesses” (Scheper-Hughes, 1995) and structural diagnosticians (Seymour, et al., 2018) can too easily fade into another form of carceral humanism unless these acts are anchored by a praxis of liberatory solidarity (Levenson and Samra, this issue) that orients clinical caregiving firmly toward abolitionist horizons. The authors may even go as far as to be suggesting that accompaniment, as a form of practice that results from a rigorous biosocial analysis within the social medicine tradition, demands (ac)complicity.

At a moment when “exploring models of care oriented towards liberation feels ever more urgent” (Karlin & Hodge, this issue), this series offer us new tools for thinking with and from a complicity consciousness “alongside others to imagine, perceive, or dwell in a possible otherwise” (Meek & Morales, 2022). Despite (and perhaps because of) the “the hope attendant in the figure of the [clinician]-anthropologist” for the discipline of social medicine (Franklin & Munyikwa, 2021), clinician-ethnographers should conceive of themselves as “negative workers” (Basaglia et al., 1987) that practice refusal when necessary and create “openings” in clinical institutions that permit emancipatory practices (Watkins, 2019). How could and should autonomous and decentralized models of care like “the Hotline” (Karlin & Hodge, this issue) be applied to other medical issues entangled with the carceral neoliberal medical industrial complex? What are clinician-ethnographers’ roles and responsibilities in building, studying, and sustaining these efforts with others? Drawing from Levenson and Samra’s collaborations with local community organizers that showed “how health workers could play a role, beyond the clinic, in changing the definitions of the state’s function and capacity,” how can clinical ethnography and social medicine better promote “collective structural competency” (Piñones-Rivera et al., 2023) and the radical reimaginations it makes possible among clinicians, social scientists, and collectives of organized patients and community members? The answers to these questions turn on whether clinical ethnography can move beyond *witnessing* structural violence toward a rethinking of health systems: defining “affirmative visions” of what could be done “in place of institutions of organized violence” (Levenson & Samra, this issue; Farmer et al., 2006).

Ultimately, these papers renew the task of social medicine in the 21st century, as a field which must move beyond the essential and established work of structural analysis toward “scholarship in solidarity with transforming worlds” (Meek & Morales, 2022), a deliberate (ac)complicity in “convoking “(Haiven & Khasnabish, 2010) and enabling abolitionist visions that uproot unjust social orderings. Structural attunement and robust social science training is not enough to interrupt or even mitigate harm within spaces of structural violence; the clinician-ethnographers in this series are generously vulnerable and reflexive about the limits of their attempts at care and therapeutic presence, describing how attempts at pragmatic accompaniment give way to carceral humanism. What is needed instead is to use the insights from clinical ethnography as a methodology of social medicine to develop a path for healthcare delivery as a form of liberatory solidarity (Dubal et al., 2021; McTighe & Raschig, 2019). This special issue begins to map some of those possibilities and where they might take us.
